# Theoretical framework and inference for fitting extreme data through the modified Weibull distribution in a first-failure censored progressive approach

**DOI:** 10.1016/j.heliyon.2024.e34418

**Published:** 2024-07-14

**Authors:** Mohamed S. Eliwa, Laila A. Al-Essa, Amr M. Abou-Senna, Mahmoud El-Morshedy, Rashad M. EL-Sagheer

**Affiliations:** a Department of Statistics and Operations Research, College of Science, Qassim University, Saudi Arabia; b Department of Statistics and Computer Science, Faculty of Science, Mansoura University, Mansoura 35516, Egypt; c Department of Mathematical Sciences, College of Science, Princess Nourah bint Abdulrahman University, P.O.Box 84428, Riyadh 11671, Saudi Arabia; d Mathematics Department, Faculty of Engineering, Shoubra, Benha University, Cairo, Egypt; e Department of Mathematics, Harbin Institute of Technology, Harbin 150001, China; f Department of Mathematics, College of Science and Humanities in Al-Kharj, Prince Sattam bin Abdulaziz University, Al-Kharj 11942, Saudi Arabia; g Department of Mathematics, Faculty of Science, Mansoura University, Mansoura 35516, Egypt; h Mathematics Department, Faculty of Science, Al-Azhar University, Naser city 11884, Cairo, Egypt; i High Institute of Computer and Management Information Systems, First Statement, New Cairo 11865, Cairo, Egypt

**Keywords:** Statistical model, Censored data, Bayesian approach, Markov chain Monte Carlo, Simulation, Data analysis

## Abstract

The importance of biomedical physical data is underscored by its crucial role in advancing our comprehension of human health, unraveling the mechanisms underlying diseases, and facilitating the development of innovative medical treatments and interventions. This data serves as a fundamental resource, empowering researchers, healthcare professionals, and scientists to make informed decisions, pioneer research, and ultimately enhance global healthcare quality and individual well-being. It forms a cornerstone in the ongoing pursuit of medical progress and improved healthcare outcomes. This article aims to tackle challenges in estimating unknown parameters and reliability measures related to the modified Weibull distribution when applied to censored progressive biomedical data from the initial failure occurrence. In this context, the article proposes both classical and Bayesian techniques to derive estimates for unknown parameters, survival, and failure rate functions. Bayesian estimates are computed considering both asymmetric and symmetric loss functions. The Markov chain Monte Carlo method is employed to obtain these Bayesian estimates and their corresponding highest posterior density credible intervals. Due to the inherent complexity of these estimators, which cannot be theoretically compared, a simulation study is conducted to evaluate the performance of various estimation procedures. Additionally, a range of optimization criteria is utilized to identify the most effective progressive control strategies. Lastly, the article presents a medical application to illustrate the effectiveness of the proposed estimators. Numerical findings indicate that Bayesian estimates outperform other estimation methods by achieving minimal root mean square errors and narrower interval lengths.

## 1 Introduction

Effectively analyzing biomedical physical data holds the potential to advance personalized medicine, contribute to disease prevention, enable early diagnosis, and support the development of targeted therapies. However, it also introduces notable ethical and privacy considerations, particularly when handling sensitive patient information. Collaboration between researchers and healthcare professionals becomes imperative to ensure the responsible and secure management of biomedical data while leveraging its potential for improving human health. In various experimental and statistical scenarios, obtaining comprehensive information on failure units can be exceedingly challenging, if not impossible, due to constraints such as cost and time limitations. This challenge is particularly pertinent in reliability research, medical survival analysis, and industrial life testing trials, where minimizing total testing duration and associated high costs is of utmost importance. In these experiments, units may either fail or be removed before failing, and these removed units may be utilized in subsequent experiments. As a result, censorship occurs when the precise ages of the units in the test are known. Currently, control and censorship methodologies encompass various types that have been implemented in lifetime experiments. Among these, one of the most commonly used methods is Type II censoring, where all *n* units are initially included in the test, and the test concludes when the pre-determined m−th unit (1≤m≤n) fails. Furthermore, the time at which the test ends is random.

Despite the potential for extended testing times due to the presence of units with high ages, many experimenters opt for Type II censorship. However, it's worth noting that Type II censorship has a drawback in that units cannot be withdrawn from the test once initiated (see Kundu and Howlader [1], Balakrishnan and Han [2] and Lawless [3]). Hence, a censorship method that offers more flexibility compared to Type II censorship, allowing for the withdrawal of units during the test's duration, is known as Type II progressive censorship (PT2C). Progressive control strategies have garnered significant attention in recent times due to their adaptability in permitting units to be removed at any point other than the endpoint. Progressive control and censorship systems have been introduced in various forms, including Type I, Type II, and hybrid progressive control systems. However, it's worth noting that conducting investigations, especially when dealing with highly reliable products, can be time-consuming using these control methods. A robust solution to this issue involves grouping the tested units into several sets, each containing an equal number of units. The time until the first failure within each group is recorded, resulting in what is known as a progressive first failure control chart. This approach has gained popularity in recent years for reliability analysis and life testing studies. For more details on estimation based on PT2C with applications, see EL-Sagheer [4], Wu and Gui [5], EL-Sagheer et al. [6], Khodadadian et al. [7], Noii et al. [8], Khodadadian et al. [9], Khodadadian et al. [10] and Luo et al. [11].

**Figure 1:**
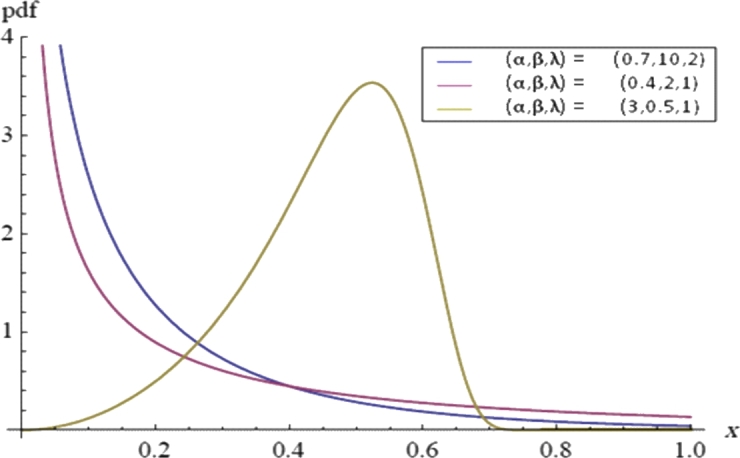
The PDFs of the MWD with different *α*, *β* and *λ*.

**Figure 2:**
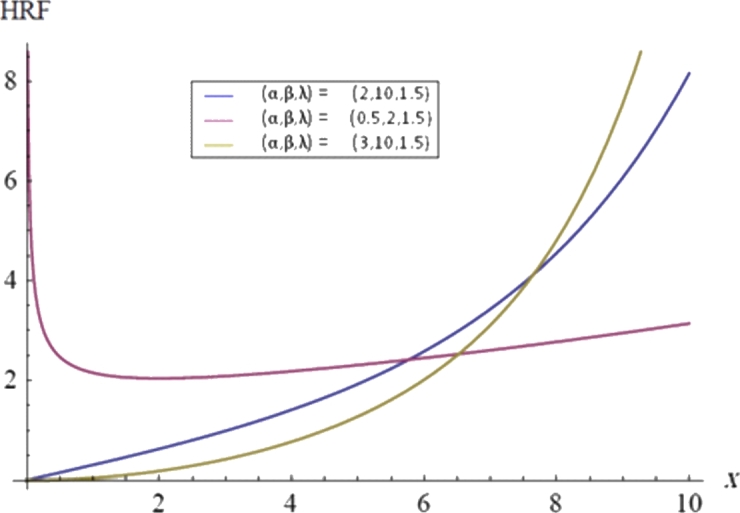
The HRFs of the MWD with different *α*, *β* and *λ*.

Although PT2C can enhance experimental efficiency, the testing duration remains a concern. Johnson [12] introduced a life test method where test units are grouped and all groups are tested simultaneously until the first failure occurs in each group. This type of censoring is known as first-failure censoring (FFC), as discussed by Wu et al. [13] and Wu and Yu [14]. Unfortunately, once grouped, units cannot be removed during FFC. To address this limitation and further improve test efficiency, Wu and Kus [15] proposed a new life test method that combines PT2C with FFC, termed progressive first failure censoring (PFFC). PFFC allows for the removal of certain groups of test units before observing any failures in those groups. Many researchers have explored statistical inference using PFFC across various models, see for instance, Ahmadi and Doostparast [16], Kayal et al. [17], Shi and Shi [18] and EL-Sagheer et al. [19]. In this paper, the modified Weibull distribution (MWD) is discussed based on the PFFC approach. Xie et al. [20] suggested the MWD as a generalization of the WD. Moreover, the statistical properties and detailed statistical analysis were given in Tang et al. [21] and Chen [22]. If *X* follows a MWD, then the probability (PDF), cumulative (CDF), survival (SF), hazard rate (HRF) and inverse hazard rate functions (IHRF) are given, respectively, as

$$f(x)=\lambda \alpha {\left(\frac{x}{\beta }\right)}^{\alpha -1}\exp\left\{{\left(\frac{x}{\beta }\right)}^{\alpha }+\lambda \beta \left(1-\exp\left[{\left(\frac{x}{\beta }\right)}^{\alpha }\right]\right)\right\},\;x>0,\;\alpha ,\beta ,\lambda >0,$$

$$F(x)=1-\exp\left\{\lambda \beta \left(1-\exp\left\{{\left(\frac{x}{\beta }\right)}^{\alpha }\right\}\right)\right\},\;x>0,$$

$$S(t)=\exp\left\{\lambda \beta \left(1-\exp\left[{\left(\frac{t}{\beta }\right)}^{\alpha }\right]\right)\right\},\;t>0,$$

$$h(t)=\lambda \alpha {\left(\frac{t}{\beta }\right)}^{\alpha -1}\exp\left\{{\left(\frac{t}{\beta }\right)}^{\alpha }\right\},$$

and

$$r\left(t\right)=\frac{\lambda \alpha {\left(\frac{t}{\beta }\right)}^{\alpha -1}\exp\left\{{\left(\frac{t}{\beta }\right)}^{\alpha }+\lambda \beta \left(1-\exp\left\{{\left(\frac{t}{\beta }\right)}^{\alpha }\right\}\right)\right\}}{1-\exp\left\{\lambda \beta \left(1-\exp\left\{{\left(\frac{t}{\beta }\right)}^{\alpha }\right\}\right)\right\}},$$

where *λ* is the scale parameter and both *α* and *β* are the shape parameters. It is clear that the exponential power distribution EPD(α,β) is a special case of the MWD with λ=1, see Smith and Bain [23], Aarset [24] and Gupta et al. [25]. Also, the shape of HRF of the MWD depends only on the shape parameter *α* as follows. For α≥1, the HRF is increasing function. For 0<α<1, the HRF is decreasing for $x<\frac{\beta }{\alpha }\left(\frac{1}{\alpha }-1\right)$ and increasing for $x<\frac{\beta }{\alpha }\left(\frac{1}{\alpha }-1\right)$. The PDF and HRF plots of the MWD are given in Figure 1, Figure 2, respectively.

The main aim of the article is to address the challenges associated with estimating unknown parameters and reliability measures when applying the MWD to censored progressive biomedical data. Specifically, the article aims to: Develop and compare classical and Bayesian techniques for parameter estimation, survival, and failure rate functions under the modified Weibull distribution framework. Compute Bayesian estimates using both asymmetric and symmetric loss functions, employing the MCMC method to derive these estimates and their corresponding credible intervals. Conduct a simulation study to evaluate the performance of the proposed estimation procedures, considering various optimization criteria to identify optimal progressive control strategies. Demonstrate the practical application of the proposed estimators through a medical case study, showcasing their effectiveness in biomedical data analysis. Provide numerical evidence supporting the superiority of Bayesian estimates, showing reduced mean square errors and narrower interval lengths compared to alternative estimation methods. In essence, the article seeks to contribute methodological advancements in statistical inference for extreme biomedical data, particularly in the context of first-failure censored progressive scenarios, thereby enhancing the reliability and applicability of statistical methods in medical research and healthcare quality improvement efforts.

The paper layout is arranged as follows. Section 2 shows the maximum likelihood estimates (MLEs) and observed Fisher information matrix (FIM). Bayes estimates are obtained using Lindley's and Markov chain Monte Carlo (MCMC) approaches in Section 3. In Section 4, a simulation study is carried out. Application on renal transplant survival times is studied in Section 5. Finally, the article is summed up in Section 6.

## 2 MLE

Maximum Likelihood Estimation (MLE) stands as a cornerstone in statistical inference, offering a powerful framework to deduce parameters that best describe the underlying data distribution. By maximizing the likelihood function, MLE seeks to find the values of parameters that make the observed data most probable under the assumed statistical model. Widely employed across disciplines from finance to biology, MLE facilitates robust parameter estimation for complex models, relying on the assumption of independently and identically distributed (i.i.d.) data. This methodological approach inherently balances simplicity with efficiency, providing optimal estimates under ideal conditions of large sample sizes. Understanding MLE empowers researchers and practitioners to make informed decisions based on data-driven insights, essential in shaping modern scientific and industrial practices.

This section discusses the MLE given some observed data. We have extended the Weibull distribution to have 3-parameter as a sort of model complexity for attaining better fitting of the data and achieving a high level of accuracy. Furthermore, we compute the estimate and the approximate confidence intervals for the survival function (SF), hazard rate function (HRF), and inverse hazard rate function (IHRF) which, to the best of our knowledge, have not been discussed a lot in the literature. Let $X_{i:m:n:k}^{R}$; i=1,2,...,m, be the PFFC sample from MWD with censoring scheme *R*. The joint probability density function of $X_{1:m:n:k}^{R}<X_{2:m:n:k}^{R}<...<X_{m:m:n:k}^{R}$ is given by

$$f_{1,2,...,m}\left(X_{1:m:n:k}^{R},X_{2:m:n:k}^{R},...,X_{m:m:n:k}^{R}\right)=Dk^{m}\prod_{i=1}^{m}f\left(X_{i:m:n:k}^{R}\right){\left(1-F\left(X_{i:m:n:k}^{R}\right)\right)}^{k\left(R_{i}+1\right)-1},$$

where

$$D=n\left(n-1-R_{1}\right)\left(n-1-R_{1}-R_{2}\right)...\left(n-m+1-R_{1}-R_{2}-...R_{m-1}\right).$$

For more details on the model description see Wu and Kus [15]. Thus, the log-likelihood function without normalized constant can be expressed as

(1) $$L(\alpha ,\beta ,\lambda |\underline{x})\propto m\log\left(\alpha \lambda \right)+\left(\alpha -1\right)\sum_{i=1}^{m}\log\left(\frac{x_{i}}{\beta }\right)+\sum_{i=1}^{m}\left(\frac{x_{i}}{\beta }\right)+kn\lambda \beta -\sum_{i=1}^{m}kn\lambda \beta \left(1+R_{i}\right)\exp\left\{{\left(\frac{x_{i}}{\beta }\right)}^{\alpha }\right\}.$$

It is possible to get the maximum likelihood (ML) estimators by solving the following likelihood equations after setting the partial derivatives of Eq. (1) with respect to *α*, *β*, and *λ* to zero where

(2) $$\frac{m}{\alpha }+\sum_{i=1}^{m}\left(\frac{x_{i}}{\beta }\right)+\sum_{i=1}^{m}{\left(\frac{x_{i}}{\beta }\right)}^{\alpha }\log\left(\frac{x_{i}}{\beta }\right)\left(1-\lambda k\beta \left(1+R_{i}\right)\exp\left\{{\left(\frac{x_{i}}{\beta }\right)}^{\alpha }\right\}\right)=0,$$

(3) $$\frac{m\left(1-\alpha \right)}{\beta }+kn\lambda +\sum_{i=1}^{m}{\left(\frac{x_{i}}{\beta }\right)}^{\alpha }\left(\lambda \alpha k\left(1+R_{i}\right)\exp\left\{{\left(\frac{x_{i}}{\beta }\right)}^{\alpha }\right\}-\frac{\alpha }{\beta }\right)=0,$$

and

(4) $$\frac{m}{\lambda }+kn\beta -\sum_{i=1}^{m_{1}}\beta k\left(1+R_{1i}\right)\exp\left\{{\left(\frac{x_{i}}{\beta }\right)}^{\alpha }\right\}=0.$$

As the non-linear Eqs. (2), (3) and (4) are evidently unsolvable analytically, a numerical approach like Newton-Raphson is employed, as stated in EL-Sagheer [4]. Additionally, the MLE of S(t), h(t) and r(t) can be written as

$$\overset{ˆ}{S}(t)=\exp\left\{\overset{ˆ}{\lambda }\overset{ˆ}{\beta }\left(1-\exp\left[{\left(\frac{t}{\overset{ˆ}{\beta }}\right)}^{\overset{ˆ}{\alpha }}\right]\right)\right\},$$

$$\overset{ˆ}{h}\left(t\right)=\overset{ˆ}{\lambda }\overset{ˆ}{\alpha }{\left(\frac{t}{\overset{ˆ}{\beta }}\right)}^{\overset{ˆ}{\alpha }-1}\exp\left\{{\left(\frac{t}{\overset{ˆ}{\beta }}\right)}^{\overset{ˆ}{\alpha }}\right\},$$

and

$$\overset{ˆ}{r}\left(t\right)=\frac{\overset{ˆ}{\lambda }\overset{ˆ}{\alpha }{\left(\frac{t}{\overset{ˆ}{\beta }}\right)}^{\overset{ˆ}{\alpha }-1}\exp\left\{{\left(\frac{t}{\overset{ˆ}{\beta }}\right)}^{\overset{ˆ}{\alpha }}+\overset{ˆ}{\lambda }\overset{ˆ}{\beta }\left(1-\exp\left\{{\left(\frac{t}{\overset{ˆ}{\beta }}\right)}^{\overset{ˆ}{\alpha }}\right\}\right)\right\}}{1-\exp\left\{\overset{ˆ}{\lambda }\overset{ˆ}{\beta }\left(1-\exp\left\{{\left(\frac{t}{\overset{ˆ}{\beta }}\right)}^{\overset{ˆ}{\alpha }}\right\}\right)\right\}}.$$

### 2.1 Approximate confidence intervals

Approximate Confidence Intervals (ACIs), leveraging the Fisher Information Matrix (FIM), offer a robust statistical tool for estimating parameter uncertainties. By utilizing the second derivative of the log-likelihood function, FIM provides a framework to calculate ACIs efficiently. These intervals are valuable in scenarios where exact solutions are impractical, providing reliable estimates with manageable computational effort. ACIs derived from FIM enhance decision-making by quantifying the precision of parameter estimates in statistical inference. This approach is widely applied across disciplines for its versatility and reliability in uncertainty quantification. Based on the asymptotic normality of the MLEs, the ACIs of the parameters *α*, *β* and *λ* can be constructed via asymptotic variances that can be acquired from the inverse of the FIM $I^{-1}\left(\alpha ,\beta ,\lambda \right)$. Practically, we usually estimate $I^{-1}\left(\alpha ,\beta ,\lambda \right)$ by $I^{-1}\left(\overset{ˆ}{\alpha },\overset{ˆ}{\beta },\overset{ˆ}{\lambda }\right)$. Furthermore, using the following approximation is a more straightforward and legitimate process

$$\left(\overset{ˆ}{\alpha },\overset{ˆ}{\beta },\overset{ˆ}{\lambda }\right)∼N\left(\left(\alpha ,\beta ,\lambda \right),I^{-1}\left(\overset{ˆ}{\alpha },\overset{ˆ}{\beta },\overset{ˆ}{\lambda }\right)\right).$$

Therefore, the inverse of the FIM can be determined using the likelihood equations through the following form

$$I^{-1}\left(\alpha ,\beta ,\lambda \right)={\left[E\left(\begin{array}{ccc} -\frac{\partial^{2}L(\alpha ,\beta ,\lambda |\underline{x})}{\partial \alpha^{2}}\; & -\frac{\partial^{2}L(\alpha ,\beta ,\lambda |\underline{x})}{\partial \alpha \partial \beta }\; & -\frac{\partial^{2}L(\alpha ,\beta ,\lambda |\underline{x})}{\partial \alpha \partial \lambda }\\ \frac{-\partial^{2}L(\alpha ,\beta ,\lambda |\underline{x})}{\partial \beta \partial \alpha }\; & -\frac{\partial^{2}L(\alpha ,\beta ,\lambda |\underline{x})}{\partial \beta^{2}}\; & -\frac{\partial^{2}L(\alpha ,\beta ,\lambda |\underline{x})}{\partial \beta \partial \lambda }\\ -\frac{\partial^{2}L(\alpha ,\beta ,\lambda |\underline{x})}{\partial \lambda \partial \alpha }\; & -\frac{\partial^{2}L(\alpha ,\beta ,\lambda |\underline{x})}{\partial \lambda \partial \beta }\; & -\frac{\partial^{2}L(\alpha ,\beta ,\lambda |\underline{x})}{\partial \lambda^{2}} \end{array}\right)\right]}^{-1},$$

where

$$\frac{\partial^{2}L}{\partial \alpha^{2}}=\frac{-m}{\alpha^{2}}+\sum_{i=1}^{m}{\left(\frac{x_{i}}{\beta }\right)}^{\alpha }\log^{2}\left(\frac{x_{i}}{\beta }\right)\left(1-\lambda k\beta \left(1+R_{i}\right)\left(1-\frac{x_{i}}{\beta }\right)\exp\left\{{\left(\frac{x_{i}}{\beta }\right)}^{\alpha }\right\}\right),$$

$$\frac{\partial^{2}L}{\partial \beta^{2}}=\frac{m\left(\alpha -1\right)}{\beta^{2}}+\sum_{i=1}^{m}{\left(\frac{x_{i}}{\beta }\right)}^{\alpha }\frac{\alpha }{\beta }\left(\frac{1}{\beta }\left(1+\alpha \right)-\lambda k\beta \left(1+R_{i}\right)\left(1-\frac{x_{i}}{\beta }\right)\exp\left\{{\left(\frac{x_{i}}{\beta }\right)}^{\alpha }\right\}\right),$$

$$\frac{\partial^{2}L}{\partial \lambda^{2}}=\frac{-m}{\lambda^{2}}.$$

Therefore, (1−γ)100% ACIs for parameters α,β and *λ* become

$$\overset{ˆ}{\alpha }\pm Z_{\gamma /2}\sqrt{Var(\overset{ˆ}{\alpha })},\;\overset{ˆ}{\beta }\pm Z_{\gamma /2}\sqrt{Var(\overset{ˆ}{\beta })},\;\overset{ˆ}{\lambda }\pm Z_{\gamma /2}\sqrt{Var(\overset{ˆ}{\lambda })},$$

where $Z_{\gamma /2}$ is the percentile of the standard normal distribution with right-tail probability γ/2. According to delta method discussed in Greene [26], the variances of $\overset{ˆ}{S}\left(t\right)$, $\overset{ˆ}{h}\left(t\right)$ and $\overset{ˆ}{r}\left(t\right)$ can be roughly calculated using

$$\tau_{\overset{ˆ}{S}\left(t\right)}^{2}={\left[△\overset{ˆ}{S}(t)\right]}^{T}[I^{-1}][△\overset{ˆ}{S}(t)],$$

$$\tau_{\overset{ˆ}{h}\left(t\right)}^{2}={\left[△\overset{ˆ}{h}(t)\right]}^{T}[I^{-1}][△\overset{ˆ}{h}(t)],$$

and

$$\tau_{\overset{ˆ}{r}\left(t\right)}^{2}={\left[△\overset{ˆ}{r}(t)\right]}^{T}[I^{-1}][△\overset{ˆ}{r}(t)],$$

where $△\overset{ˆ}{S}\left(t\right)$, $△\overset{ˆ}{h}\left(t\right)$ and $△\overset{ˆ}{r}\left(t\right)$ are the gradient of $\overset{ˆ}{S}\left(t\right)$, $\overset{ˆ}{h}\left(t\right)$ and $\overset{ˆ}{r}\left(t\right)$ with respect to *α*, *β* and *λ*. Therefore, (1−γ)100% ACIs for S(t), h(t) and r(t) are

$$\overset{ˆ}{S}\left(t\right)\pm Z_{\gamma /2}\sqrt{\tau_{\overset{ˆ}{S}\left(t\right)}^{2}},\;\overset{ˆ}{h}\left(t\right)\pm Z_{\gamma /2}\sqrt{\tau_{\overset{ˆ}{h}\left(t\right)}^{2}},\;\overset{ˆ}{r}\left(t\right)\pm Z_{\gamma /2}\sqrt{\tau_{\overset{ˆ}{r}\left(t\right)}^{2}}.$$

## 3 Bayesian estimation

Bayesian estimation represents a powerful paradigm in statistical inference, rooted in Bayes' theorem, which updates prior beliefs with observed data to yield posterior distributions. Unlike frequentist methods, Bayesian estimation incorporates prior knowledge into the analysis, making it particularly adept in scenarios with limited data or when historical information is available. This approach allows for the quantification of uncertainty through posterior distributions, offering a comprehensive understanding of parameter estimates. Bayesian methods excel in complex modeling tasks, where incorporating prior information can enhance accuracy and robustness. The gamma distribution (GD) family is widely recognized for its flexibility in accommodating a diverse range of prior beliefs held by experimenters. Moreover, statisticians are particularly drawn to its richness; adjusting its parameters yields new data that introduces fresh insights. As a result, the GD garners significant attention within the statistical community. Here, it is considered that the parameters *α*, *β* and *λ* are independent and follow gamma distributions

$$\pi (\alpha )\propto \alpha^{a_{1}-1}\exp\left\{-b_{1}\alpha \right\},\pi (\beta )\propto \beta^{a_{2}-1}\exp\left\{-b_{2}\beta \right\},$$

and

$$\pi (\lambda )\propto \lambda^{a_{3}-1}\exp\left\{-b_{3}\lambda \right\},$$

where, the hyperparameters $a_{i}$ and $b_{i\;}$, i=1, 2 and 3 reflect the knowledge of prior about (α,β,λ) and assumed to be nonnegative and known. A special case: When all hyperparameters of GD are zero, we obtain Jaffrey prior in the form $\frac{1}{\alpha },\frac{1}{\beta }$ and $\frac{1}{\lambda }$. Therefore, the joint prior can be expressed by

π(α,β,λ)∝π(α)π(β)π(λ).

Consequently, via Bayes' theorem, the joint posterior is

$$\pi^{⁎}(\alpha ,\beta ,\lambda |\underline{x})=\frac{\ell (\alpha ,\beta ,\lambda |\underline{x})\times \pi (\alpha ,\beta ,\lambda )}{\int_{0}^{\infty }\int_{0}^{\infty }\int_{0}^{\infty }\ell (\alpha ,\beta ,\lambda |\underline{x})\times \pi (\alpha ,\beta ,\lambda )d\alpha d\beta d\lambda }.$$

Therefore, the Bayes estimator for any function say g(α,β,λ), under squared error loss function (SELF) is

(5) $$\overset{ˆ}{g}(\alpha ,\beta ,\lambda )=E_{\alpha ,\beta ,\lambda |x}(g(\alpha ,\beta ,\lambda ))=\frac{\int_{0}^{\infty }\int_{0}^{\infty }\int_{0}^{\infty }g(\alpha ,\beta ,\lambda )\times \ell (\alpha ,\beta ,\lambda |x)\times \pi (\alpha ,\beta ,\lambda )d\alpha d\beta d\lambda }{\int_{0}^{\infty }\int_{0}^{\infty }\int_{0}^{\infty }\ell (\alpha ,\beta ,\lambda |x)\times \pi (\alpha ,\beta ,\lambda )d\alpha d\beta d\lambda },$$

while, the Bayes estimator for g(α,β,λ) under general entropy loss function (GELF) is

(6) $${\overset{ˆ}{g}}_{BL}\left(\alpha ,\beta ,\lambda \right)={\left(E_{\alpha ,\beta ,\lambda |data}\left[{\left(g(\alpha ,\beta ,\lambda )\right)}^{-\epsilon }\right]\right)}^{\frac{-1}{\epsilon }},\;\;\varepsilon \neq 0\;,$$

and

(7) $$E_{\alpha ,\beta ,\lambda |data}\left[e^{-\varepsilon g\left(\alpha ,\beta ,\lambda \right)}\right]=\frac{\int_{0}^{\infty }\int_{0}^{\infty }\int_{0}^{\infty }e^{-\varepsilon g\left(\alpha ,\beta ,\lambda \right)}\times L(\alpha ,\beta ,\lambda |\underline{x})\times \pi (\alpha ,\beta ,\lambda )d\alpha d\beta d\lambda }{\int_{0}^{\infty }\int_{0}^{\infty }\int_{0}^{\infty }L(\alpha ,\beta ,\lambda |\underline{x})\times \pi (\alpha ,\beta ,\lambda )d\alpha d\beta d\lambda }\;.$$

It is not possible to compute Eqs. (5), (6), and (7) analytically. Therefore, Lindley approximation and MCMC technique are being used to obtain the Bayes estimates for the parameters α,β and *λ*.

### 3.1 Lindley's technique

Lindley's technique offers a simplified method to calculate posterior distributions without requiring extensive computational resources. This technique approximates Bayesian inference by leveraging a second-order Taylor expansion around the mode of the prior distribution. By focusing on local behavior, Lindley's approximation provides a pragmatic solution for situations where exact posterior calculations are challenging. This method is particularly useful in scenarios where the posterior distribution is unimodal and symmetric around its mode, offering a computationally efficient alternative to more complex Bayesian inference techniques. The Lindley approximation was first presented by Lindley [27]. It is significant because it allows the Bayes estimators to be estimated in a way that doesn't require integrals, as will be demonstrated below. Let us consider

$$I=\frac{\int_{\left(\alpha ,\beta ,\lambda \right)}w\left(\alpha ,\beta ,\lambda \right)e^{\ell (\alpha ,\beta ,\lambda )+\rho \left(\alpha ,\beta ,\lambda \right)}d\left(\alpha ,\beta ,\lambda \right)}{\int_{\left(\alpha ,\beta ,\lambda \right)}e^{\ell (\alpha ,\beta ,\lambda )+\rho \left(\alpha ,\beta ,\lambda \right)}d\left(\alpha ,\beta ,\lambda \right)},$$

where w(α,β,λ) is a function of *α*, *β* or *λ* and ρ(α,β,λ)=log⁡π(α,β,λ). Then the ratio of the two integrals can be calculated as follows

(8) $$I=w\left(\overset{ˆ}{\alpha },\overset{ˆ}{\beta },\overset{ˆ}{\lambda }\right)+{\overset{ˆ}{w}}_{1}{\overset{ˆ}{a}}_{1}+{\overset{ˆ}{w}}_{2}{\overset{ˆ}{a}}_{2}+{\overset{ˆ}{w}}_{3}{\overset{ˆ}{a}}_{3}+{\overset{ˆ}{a}}_{4}+{\overset{ˆ}{a}}_{5}+\overset{ˆ}{A}\left({\overset{ˆ}{w}}_{1}\tau_{11}+{\overset{ˆ}{w}}_{2}\tau_{12}+{\overset{ˆ}{w}}_{3}\tau_{13}\right)+\overset{ˆ}{B}\left({\overset{ˆ}{w}}_{1}\tau_{21}+{\overset{ˆ}{w}}_{2}\tau_{22}+{\overset{ˆ}{w}}_{3}\tau_{23}\right)+\overset{ˆ}{C}\left({\overset{ˆ}{w}}_{1}\tau_{31}+{\overset{ˆ}{w}}_{2}\tau_{32}+{\overset{ˆ}{w}}_{3}\tau_{33}\right)\;,$$

where,

$${\overset{ˆ}{a}}_{i}={\overset{ˆ}{\rho }}_{1}\tau_{i1}+{\overset{ˆ}{\rho }}_{2}\tau_{i2}+{\overset{ˆ}{\rho }}_{3}\tau_{i3};\;\;i=1,2,3,$$

$${\overset{ˆ}{a}}_{4}=\tau_{12}{\overset{ˆ}{w}}_{12}+\tau_{13}{\overset{ˆ}{w}}_{13}+\tau_{23}{\overset{ˆ}{w}}_{23}\;,$$

$${\overset{ˆ}{a}}_{5}=\frac{1}{2}(\tau_{11}{\overset{ˆ}{w}}_{11}+\tau_{22}{\overset{ˆ}{w}}_{22}+\tau_{33}{\overset{ˆ}{w}}_{33})\;,$$

$$\overset{ˆ}{A}=\tau_{11}{\overset{ˆ}{\ell }}_{111}+2\tau_{12}{\overset{ˆ}{\ell }}_{121}+2\tau_{13}{\overset{ˆ}{\ell }}_{131}+2\tau_{23}{\overset{ˆ}{\ell }}_{231}+\tau_{22}{\overset{ˆ}{\ell }}_{221}+\;\tau_{33}{\overset{ˆ}{\ell }}_{331},$$

$$\overset{ˆ}{B}=\tau_{11}{\overset{ˆ}{\ell }}_{112}+2\tau_{12}{\overset{ˆ}{\ell }}_{122}+2\tau_{13}{\overset{ˆ}{\ell }}_{132}+2\tau_{23}{\overset{ˆ}{\ell }}_{232}+\tau_{22}{\overset{ˆ}{\ell }}_{222}+\tau_{33}{\overset{ˆ}{\ell }}_{332},$$

and

$$\overset{ˆ}{C}=\tau_{11}{\overset{ˆ}{\ell }}_{113}+2\tau_{12}{\overset{ˆ}{\ell }}_{123}+2\tau_{13}{\overset{ˆ}{\ell }}_{133}+2\tau_{23}{\overset{ˆ}{\ell }}_{233}+\tau_{22}{\overset{ˆ}{\ell }}_{223}+\tau_{33}{\overset{ˆ}{\ell }}_{333},$$

where subscripts 1, 2 and 3 on the right-hand side stand for *α*, *β* and *λ* respectively.

$${\overset{ˆ}{\rho }}_{i}={\left(\frac{\partial \rho }{\partial \Omega_{i}}\right)}_{↓\left({\overset{ˆ}{\Omega }}_{1},{\overset{ˆ}{\Omega }}_{2},{\overset{ˆ}{\Omega }}_{3}\right)};\;i\;=1,2,3,\;\;\Omega_{1}=\alpha ,\;\;\Omega_{2}=\beta ,\;\;\Omega_{3}=\lambda ,$$

$${\overset{ˆ}{w}}_{ij}={\left(\frac{\partial^{2}w\left(\Omega_{1},\Omega_{2},\Omega_{3}\right)}{\partial \Omega_{i}\partial \Omega_{j}}\right)}_{↓\left({\overset{ˆ}{\Omega }}_{1},{\overset{ˆ}{\Omega }}_{2},{\overset{ˆ}{\Omega }}_{3}\right)};\;i,j\;=1,2,3,$$

$${\overset{ˆ}{\ell }}_{ij}={\left(\frac{\partial^{2}\ell (\Omega_{1},\Omega_{2},\Omega_{3})}{\partial \Omega_{i}\partial \Omega_{j}}\right)}_{↓\left({\overset{ˆ}{\Omega }}_{1},{\overset{ˆ}{\Omega }}_{2},{\overset{ˆ}{\Omega }}_{3}\right)};\;i,j\;=1,2,3,$$

$$\tau_{ij}=\frac{-1}{{\overset{ˆ}{\ell }}_{ij}},\;i,i=1,2,3,$$

and

$${\overset{ˆ}{\ell }}_{ijk}={\left(\frac{\partial^{3}\ell (\Omega_{1},\Omega_{2},\Omega_{3})}{\partial \Omega_{i}\partial \Omega_{j}\partial \Omega_{l}}\right)}_{↓\left({\overset{ˆ}{\Omega }}_{1},{\overset{ˆ}{\Omega }}_{2},{\overset{ˆ}{\Omega }}_{3}\right)};\;i,j,l=1,2,3.$$

#### 3.1.1 Under squared error loss function

If $w\left(\overset{ˆ}{\alpha },\overset{ˆ}{\beta },\overset{ˆ}{\lambda }\right)=\overset{ˆ}{\alpha }$, $\overset{ˆ}{\beta }$, $\overset{ˆ}{\lambda }$, $\overset{ˆ}{S}\left(t\right)$, $\overset{ˆ}{h}\left(t\right)$, or $\overset{ˆ}{r}\left(t\right)$, then the Bayes estimates of *α*, *β*, *λ*, S(t), h(t) and r(t) under SELF from Eq. (8) are

$${\overset{ˆ}{\alpha }}_{Blind-SEL}=\overset{ˆ}{\alpha }+{\overset{ˆ}{w}}_{1}{\overset{ˆ}{a}}_{1}+{\overset{ˆ}{w}}_{2}{\overset{ˆ}{a}}_{2}+{\overset{ˆ}{w}}_{3}{\overset{ˆ}{a}}_{3}+{\overset{ˆ}{a}}_{4}+{\overset{ˆ}{a}}_{5}+\overset{ˆ}{A}\left({\overset{ˆ}{w}}_{1}\tau_{11}+{\overset{ˆ}{w}}_{2}\tau_{12}+{\overset{ˆ}{w}}_{3}\tau_{13}\right)+\overset{ˆ}{B}\left({\overset{ˆ}{w}}_{1}\tau_{21}+{\overset{ˆ}{w}}_{2}\tau_{22}+{\overset{ˆ}{w}}_{3}\tau_{23}\right)+\overset{ˆ}{C}\left({\overset{ˆ}{w}}_{1}\tau_{31}+{\overset{ˆ}{w}}_{2}\tau_{32}+{\overset{ˆ}{w}}_{3}\tau_{33}\right),$$

$${\overset{ˆ}{\beta }}_{Blind-SEL}=\overset{ˆ}{\beta }+{\overset{ˆ}{w}}_{1}{\overset{ˆ}{a}}_{1}+{\overset{ˆ}{w}}_{2}{\overset{ˆ}{a}}_{2}+{\overset{ˆ}{w}}_{3}{\overset{ˆ}{a}}_{3}+{\overset{ˆ}{a}}_{4}+{\overset{ˆ}{a}}_{5}+\overset{ˆ}{A}\left({\overset{ˆ}{w}}_{1}\tau_{11}+{\overset{ˆ}{w}}_{2}\tau_{12}+{\overset{ˆ}{w}}_{3}\tau_{13}\right)+\overset{ˆ}{B}\left({\overset{ˆ}{w}}_{1}\tau_{21}+{\overset{ˆ}{w}}_{2}\tau_{22}+{\overset{ˆ}{w}}_{3}\tau_{23}\right)+\overset{ˆ}{C}\left({\overset{ˆ}{w}}_{1}\tau_{31}+{\overset{ˆ}{w}}_{2}\tau_{32}+{\overset{ˆ}{w}}_{3}\tau_{33}\right),$$

$${\overset{ˆ}{\lambda }}_{Blind-SEL}=\overset{ˆ}{\lambda }+{\overset{ˆ}{w}}_{1}{\overset{ˆ}{a}}_{1}+{\overset{ˆ}{w}}_{2}{\overset{ˆ}{a}}_{2}+{\overset{ˆ}{w}}_{3}{\overset{ˆ}{a}}_{3}+{\overset{ˆ}{a}}_{4}+{\overset{ˆ}{a}}_{5}+\overset{ˆ}{A}\left({\overset{ˆ}{w}}_{1}\tau_{11}+{\overset{ˆ}{w}}_{2}\tau_{12}+{\overset{ˆ}{w}}_{3}\tau_{13}\right)+\overset{ˆ}{B}\left({\overset{ˆ}{w}}_{1}\tau_{21}+{\overset{ˆ}{w}}_{2}\tau_{22}+{\overset{ˆ}{w}}_{3}\tau_{23}\right)+\overset{ˆ}{C}\left({\overset{ˆ}{w}}_{1}\tau_{31}+{\overset{ˆ}{w}}_{2}\tau_{32}+{\overset{ˆ}{w}}_{3}\tau_{33}\right),$$

$$\overset{ˆ}{S}{\left(t\right)}_{Blind-SEL}=\overset{ˆ}{S}\left(t\right)+{\overset{ˆ}{w}}_{1}{\overset{ˆ}{a}}_{1}+{\overset{ˆ}{w}}_{2}{\overset{ˆ}{a}}_{2}+{\overset{ˆ}{w}}_{3}{\overset{ˆ}{a}}_{3}+{\overset{ˆ}{a}}_{4}+{\overset{ˆ}{a}}_{5}+\overset{ˆ}{A}\left({\overset{ˆ}{w}}_{1}\tau_{11}+{\overset{ˆ}{w}}_{2}\tau_{12}+{\overset{ˆ}{w}}_{3}\tau_{13}\right)+\overset{ˆ}{B}\left({\overset{ˆ}{w}}_{1}\tau_{21}+{\overset{ˆ}{w}}_{2}\tau_{22}+{\overset{ˆ}{w}}_{3}\tau_{23}\right)+\overset{ˆ}{C}\left({\overset{ˆ}{w}}_{1}\tau_{31}+{\overset{ˆ}{w}}_{2}\tau_{32}+{\overset{ˆ}{w}}_{3}\tau_{33}\right),$$

$$\overset{ˆ}{h}{\left(t\right)}_{Blind-SEL}=\overset{ˆ}{h}\left(t\right)+{\overset{ˆ}{w}}_{1}{\overset{ˆ}{a}}_{1}+{\overset{ˆ}{w}}_{2}{\overset{ˆ}{a}}_{2}+{\overset{ˆ}{w}}_{3}{\overset{ˆ}{a}}_{3}+{\overset{ˆ}{a}}_{4}+{\overset{ˆ}{a}}_{5}+\overset{ˆ}{A}\left({\overset{ˆ}{w}}_{1}\tau_{11}+{\overset{ˆ}{w}}_{2}\tau_{12}+{\overset{ˆ}{w}}_{3}\tau_{13}\right)+\overset{ˆ}{B}\left({\overset{ˆ}{w}}_{1}\tau_{21}+{\overset{ˆ}{w}}_{2}\tau_{22}+{\overset{ˆ}{w}}_{3}\tau_{23}\right)+\overset{ˆ}{C}\left({\overset{ˆ}{w}}_{1}\tau_{31}+{\overset{ˆ}{w}}_{2}\tau_{32}+{\overset{ˆ}{w}}_{3}\tau_{33}\right),$$

and

$$\overset{ˆ}{r}{\left(t\right)}_{Blind-SEL}=\overset{ˆ}{r}\left(t\right)+{\overset{ˆ}{w}}_{1}{\overset{ˆ}{a}}_{1}+{\overset{ˆ}{w}}_{2}{\overset{ˆ}{a}}_{2}+{\overset{ˆ}{w}}_{3}{\overset{ˆ}{a}}_{3}+{\overset{ˆ}{a}}_{4}+{\overset{ˆ}{a}}_{5}+\overset{ˆ}{A}\left({\overset{ˆ}{w}}_{1}\tau_{11}+{\overset{ˆ}{w}}_{2}\tau_{12}+{\overset{ˆ}{w}}_{3}\tau_{13}\right)+\overset{ˆ}{B}\left({\overset{ˆ}{w}}_{1}\tau_{21}+{\overset{ˆ}{w}}_{2}\tau_{22}+{\overset{ˆ}{w}}_{3}\tau_{23}\right)+\overset{ˆ}{C}\left({\overset{ˆ}{w}}_{1}\tau_{31}+{\overset{ˆ}{w}}_{2}\tau_{32}+{\overset{ˆ}{w}}_{3}\tau_{33}\right).$$

#### 3.1.2 Under general entropy loss function

If $w\left(\overset{ˆ}{\alpha },\overset{ˆ}{\beta },\overset{ˆ}{\lambda }\right)={\overset{ˆ}{\alpha }}^{-\epsilon },{\overset{ˆ}{\beta }}^{-\epsilon },{\overset{ˆ}{\lambda }}^{-\epsilon },{\left(\overset{ˆ}{S}\left(t\right)\right)}^{-\epsilon },{\left(\overset{ˆ}{h}\left(t\right)\right)}^{-\epsilon }$ or ${\left(\overset{ˆ}{r}\left(t\right)\right)}^{-\epsilon }$, then the Bayes estimates of *α*, *β*, *λ*, S(t), h(t) and r(t) under GELF from Eq. (8) are

$${\overset{ˆ}{\alpha }}_{Blind-SEL}={\overset{ˆ}{\alpha }}^{-\epsilon }+{\overset{ˆ}{w}}_{1}{\overset{ˆ}{a}}_{1}+{\overset{ˆ}{w}}_{2}{\overset{ˆ}{a}}_{2}+{\overset{ˆ}{w}}_{3}{\overset{ˆ}{a}}_{3}+{\overset{ˆ}{a}}_{4}+{\overset{ˆ}{a}}_{5}+\overset{ˆ}{A}\left({\overset{ˆ}{w}}_{1}\tau_{11}+{\overset{ˆ}{w}}_{2}\tau_{12}+{\overset{ˆ}{w}}_{3}\tau_{13}\right)+\overset{ˆ}{B}\left({\overset{ˆ}{w}}_{1}\tau_{21}+{\overset{ˆ}{w}}_{2}\tau_{22}+{\overset{ˆ}{w}}_{3}\tau_{23}\right)+\overset{ˆ}{C}\left({\overset{ˆ}{w}}_{1}\tau_{31}+{\overset{ˆ}{w}}_{2}\tau_{32}+{\overset{ˆ}{w}}_{3}\tau_{33}\right),$$

$${\overset{ˆ}{\beta }}_{Blind-SEL}={\overset{ˆ}{\beta }}^{-\epsilon }+{\overset{ˆ}{w}}_{1}{\overset{ˆ}{a}}_{1}+{\overset{ˆ}{w}}_{2}{\overset{ˆ}{a}}_{2}+{\overset{ˆ}{w}}_{3}{\overset{ˆ}{a}}_{3}+{\overset{ˆ}{a}}_{4}+{\overset{ˆ}{a}}_{5}+\overset{ˆ}{A}\left({\overset{ˆ}{w}}_{1}\tau_{11}+{\overset{ˆ}{w}}_{2}\tau_{12}+{\overset{ˆ}{w}}_{3}\tau_{13}\right)+\overset{ˆ}{B}\left({\overset{ˆ}{w}}_{1}\tau_{21}+{\overset{ˆ}{w}}_{2}\tau_{22}+{\overset{ˆ}{w}}_{3}\tau_{23}\right)+\overset{ˆ}{C}\left({\overset{ˆ}{w}}_{1}\tau_{31}+{\overset{ˆ}{w}}_{2}\tau_{32}+{\overset{ˆ}{w}}_{3}\tau_{33}\right),$$

$${\overset{ˆ}{\lambda }}_{Blind-SEL}={\overset{ˆ}{\lambda }}^{-\epsilon }+{\overset{ˆ}{w}}_{1}{\overset{ˆ}{a}}_{1}+{\overset{ˆ}{w}}_{2}{\overset{ˆ}{a}}_{2}+{\overset{ˆ}{w}}_{3}{\overset{ˆ}{a}}_{3}+{\overset{ˆ}{a}}_{4}+{\overset{ˆ}{a}}_{5}+\overset{ˆ}{A}\left({\overset{ˆ}{w}}_{1}\tau_{11}+{\overset{ˆ}{w}}_{2}\tau_{12}+{\overset{ˆ}{w}}_{3}\tau_{13}\right)+\overset{ˆ}{B}\left({\overset{ˆ}{w}}_{1}\tau_{21}+{\overset{ˆ}{w}}_{2}\tau_{22}+{\overset{ˆ}{w}}_{3}\tau_{23}\right)+\overset{ˆ}{C}\left({\overset{ˆ}{w}}_{1}\tau_{31}+{\overset{ˆ}{w}}_{2}\tau_{32}+{\overset{ˆ}{w}}_{3}\tau_{33}\right),$$

$$\overset{ˆ}{S}{\left(t\right)}_{GEL}={\left(\overset{ˆ}{S}\left(t\right)\right)}^{-\epsilon }+{\overset{ˆ}{w}}_{1}{\overset{ˆ}{a}}_{1}+{\overset{ˆ}{w}}_{2}{\overset{ˆ}{a}}_{2}+{\overset{ˆ}{w}}_{3}{\overset{ˆ}{a}}_{3}+{\overset{ˆ}{a}}_{4}+{\overset{ˆ}{a}}_{5}+\overset{ˆ}{A}\left({\overset{ˆ}{w}}_{1}\tau_{11}+{\overset{ˆ}{w}}_{2}\tau_{12}+{\overset{ˆ}{w}}_{3}\tau_{13}\right)+\overset{ˆ}{B}\left({\overset{ˆ}{w}}_{1}\tau_{21}+{\overset{ˆ}{w}}_{2}\tau_{22}+{\overset{ˆ}{w}}_{3}\tau_{23}\right)+\overset{ˆ}{C}\left({\overset{ˆ}{w}}_{1}\tau_{31}+{\overset{ˆ}{w}}_{2}\tau_{32}+{\overset{ˆ}{w}}_{3}\tau_{33}\right),$$

$$\overset{ˆ}{h}{\left(t\right)}_{GEL}={\left(\overset{ˆ}{h}\left(t\right)\right)}^{-\epsilon }+{\overset{ˆ}{w}}_{1}{\overset{ˆ}{a}}_{1}+{\overset{ˆ}{w}}_{2}{\overset{ˆ}{a}}_{2}+{\overset{ˆ}{w}}_{3}{\overset{ˆ}{a}}_{3}+{\overset{ˆ}{a}}_{4}+{\overset{ˆ}{a}}_{5}+\overset{ˆ}{A}\left({\overset{ˆ}{w}}_{1}\tau_{11}+{\overset{ˆ}{w}}_{2}\tau_{12}+{\overset{ˆ}{w}}_{3}\tau_{13}\right)+\overset{ˆ}{B}\left({\overset{ˆ}{w}}_{1}\tau_{21}+{\overset{ˆ}{w}}_{2}\tau_{22}+{\overset{ˆ}{w}}_{3}\tau_{23}\right)+\overset{ˆ}{C}\left({\overset{ˆ}{w}}_{1}\tau_{31}+{\overset{ˆ}{w}}_{2}\tau_{32}+{\overset{ˆ}{w}}_{3}\tau_{33}\right),$$

and

$$\overset{ˆ}{r}{\left(t\right)}_{GEL}={\left(\overset{ˆ}{r}\left(t\right)\right)}^{-\epsilon }+{\overset{ˆ}{w}}_{1}{\overset{ˆ}{a}}_{1}+{\overset{ˆ}{w}}_{2}{\overset{ˆ}{a}}_{2}+{\overset{ˆ}{w}}_{3}{\overset{ˆ}{a}}_{3}+{\overset{ˆ}{a}}_{4}+{\overset{ˆ}{a}}_{5}+\overset{ˆ}{A}\left({\overset{ˆ}{w}}_{1}\tau_{11}+{\overset{ˆ}{w}}_{2}\tau_{12}+{\overset{ˆ}{w}}_{3}\tau_{13}\right)+\overset{ˆ}{B}\left({\overset{ˆ}{w}}_{1}\tau_{21}+{\overset{ˆ}{w}}_{2}\tau_{22}+{\overset{ˆ}{w}}_{3}\tau_{23}\right)+\overset{ˆ}{C}\left({\overset{ˆ}{w}}_{1}\tau_{31}+{\overset{ˆ}{w}}_{2}\tau_{32}+{\overset{ˆ}{w}}_{3}\tau_{33}\right).$$

It is known that Lindley's approximation does not make the interval estimation. So, we will construct the credible intervals (CRIs) of the unknown quantities based on MCMC technique.

### 3.2 MCMC technique

Markov chain Monte Carlo (MCMC) techniques stand as a cornerstone in Bayesian estimation, offering powerful tools to approximate complex posterior distributions through iterative sampling. Originating from the marriage of Markov chains and Monte Carlo methods, MCMC has revolutionized statistical inference by enabling practitioners to tackle high-dimensional problems that defy conventional analytical solutions. At its core, MCMC generates a sequence of correlated samples from the target distribution by constructing a Markov chain whose equilibrium distribution matches the posterior of interest. This chain's ergodicity ensures that with sufficient iterations, samples converge to the true posterior distribution, overcoming the curse of dimensionality often encountered in Bayesian inference. Several types of MCMC algorithms have emerged to address varying challenges in Bayesian estimation. The foundational Metropolis-Hastings (M-H) algorithm remains widely used, proposing candidate states based on an acceptance criterion. Its extension, the Gibbs sampler, simplifies multivariate distributions by sampling from conditionals iteratively. Both methods exemplify the adaptability of MCMC to different problem structures and data types, see Geman and Geman [28], Metropolis et al. [29] and Hastings [30]. Further innovations include the Hamiltonian Monte Carlo (HMC), which leverages gradient information to improve sampling efficiency, particularly in high-dimensional spaces. Sequential Monte Carlo (SMC) methods provide alternatives for dynamic models or scenarios with evolving data streams, ensuring robustness and adaptability in Bayesian analysis. In summary, MCMC techniques have become indispensable in Bayesian statistics, offering a principled approach to exploring and summarizing complex posterior distributions. Their evolution continues to enrich the field, enabling researchers and practitioners to extract meaningful insights from increasingly intricate datasets and models. The joint posterior density can be reformulated as follows

$$\pi^{⁎}(\alpha ,\beta ,\lambda |\underline{x})\propto \lambda^{m+a_{3}-1}\alpha^{m+a_{1}-1}\beta^{a_{2}-1}\left[\prod_{i=1}^{m}{\left(\frac{x_{i}}{\beta }\right)}^{\alpha -1}\right]\exp\left\{-b_{1}\alpha -b_{2}\beta -b_{3}\lambda \right\}\times \exp\left\{\sum_{i=1}^{m}{\left(\frac{x_{i}}{\beta }\right)}^{\alpha }+\lambda \beta k\left(1+R_{i}\right)\left(1-\exp\left\{{\left(\frac{x_{i}}{\beta }\right)}^{\alpha }\right\}\right)\right\}.$$

Thus, the conditional densities can be expressed as

(9) $$\pi_{1}^{⁎}(\alpha |\beta ,\lambda ,\underline{x})\propto \;\alpha^{m+a_{1}-1}\exp\left\{\sum_{i=1}^{m}{\left(\frac{x_{i}}{\beta }\right)}^{\alpha }+\alpha \log\left(\frac{x_{i}}{\beta }\right)-\lambda \beta k\left(1+R_{i}\right)\exp\left[{\left(\frac{x_{i}}{\beta }\right)}^{\alpha }\right]\right\}\times \exp\left\{-b_{1}\alpha \right\},$$

(10) $$\pi_{2}^{⁎}(\beta |\alpha ,\lambda ,\underline{x})\propto \beta^{a_{2}-1}\exp\left\{\sum_{i=1}^{m}{\left(\frac{x_{i}}{\beta }\right)}^{\alpha }+\left(\alpha -1\right)\log\left(\frac{x_{i}}{\beta }\right)+\lambda \beta k\left(1+R_{i}\right)\left(1-\exp\left[{\left(\frac{x_{i}}{\beta }\right)}^{\alpha }\right]\right)\right\}\times \exp\left\{-b_{2}\beta \right\},$$

and

(11) $$\pi_{3}^{⁎}(\lambda |\alpha ,\beta ,\underline{x})∼Gamma\left(m+a_{3},\sum_{i=1}^{m}\beta k\left(1+R_{i}\right)\left(\exp\left\{{\left(\frac{x_{i}}{\beta }\right)}^{\alpha }\right\}-1\right)+b_{3}\right).$$

Equation (11) follows a GD, enabling the straightforward generation of samples for *λ* using any gamma-generating routine. Conversely, Eqs (9) and (10) do not conform to established distributions, necessitating the use of MCMC techniques for sampling. Specifically, the algorithm will employ Gibbs sampling and the M-H algorithm in sequential steps to generate samples from these equations.

1. Use ${\overset{ˆ}{\alpha }}^{\left(0\right)},{\overset{ˆ}{\beta }}^{\left(0\right)}$ and ${\overset{ˆ}{\lambda }}^{\left(0\right)}$ as the initial values.
2. Set j=1.
3. Generate $\lambda^{\left(j\right)}$ from Gamma$\left(m+a_{3},\sum_{i=1}^{m}\beta k\left(1+R_{i}\right)\left(\exp\left\{{\left(\frac{x_{i}}{\beta^{\left(j-1\right)}}\right)}^{\alpha^{\left(j-1\right)}}\right\}-1\right)+b_{3}\right)$.
4. Using M-H algorithm, generate $\alpha^{\left(i\right)}$ and $\beta^{\left(i\right)}$ from $\pi_{1}^{⁎}(\alpha^{\left(j-1\right)}|\beta^{\left(j-1\right)},\lambda^{\left(j\right)},\underline{x})$ and $\pi_{2}^{⁎}(\beta^{\left(j-1\right)}|\alpha^{\left(j-1\right)},\lambda^{\left(j\right)},\underline{x})$ with $N(\alpha^{\left(j-1\right)},Var(\overset{ˆ}{\alpha }))$ and $N(\beta^{\left(j-1\right)},Var(\overset{ˆ}{\beta }))$, respectively.
  (a) Generate $\alpha^{⁎}$ from $N\left(\alpha^{\left(j-1\right)},var\left(\overset{ˆ}{\alpha }\right)\right)$ and $\beta^{⁎}$ from $N\left(\beta^{\left(j-1\right)},var\left(\overset{ˆ}{\beta }\right)\right)$.
  (b) Evaluate the probabilities
    $$Q_{\alpha }=\min\left[1,\frac{\pi_{1}^{⁎}(\alpha^{⁎}|\beta^{\left(j-1\right)},\lambda^{\left(j\right)},\underline{x})}{\pi_{1}^{⁎}(\alpha^{\left(j-1\right)}|\beta^{\left(j-1\right)},\lambda^{\left(j\right)},\underline{x})}\right],Q_{\beta }=\min\left[1,\frac{\pi_{2}^{⁎}(\beta^{⁎}|\alpha^{\left(j\right)},\lambda^{\left(j\right)},\underline{x})}{\pi_{2}^{⁎}(\beta^{\left(j-1\right)}|\alpha^{\left(j\right)},\lambda^{\left(j\right)},\underline{x})}\right],$$
  (c) Generate a $\rho_{1}$ and $\rho_{2}$ from a Uniform (0,1).
  (d) If $\rho_{1}<Q_{\alpha }$ accept the proposal and set $\alpha^{⁎}=\alpha^{\left(j\right)}$, else set $\alpha^{\left(j\right)}=\alpha^{\left(j-1\right)}$.
  (e) If $\rho_{2}<Q_{\beta }$ accept the proposal and set $\beta^{⁎}=\beta^{\left(j\right)}$, else set $\beta^{\left(j\right)}=\beta^{\left(j-1\right)}$.
5. Compute SF, HF and IHF as
  $$S^{\left(j\right)}(t)=\exp\left\{\lambda^{\left(j\right)}\beta^{\left(j\right)}\left(1-\exp\left[{\left(\frac{t}{\beta^{\left(i\right)}}\right)}^{\alpha^{\left(j\right)}}\right]\right)\right\},$$
  $$h^{\left(j\right)}(t)=\lambda^{\left(j\right)}\alpha^{\left(j\right)}{\left(\frac{t}{\beta^{\left(j\right)}}\right)}^{\alpha^{\left(j\right)}-1}\exp\left\{{\left(\frac{t}{\beta^{\left(j\right)}}\right)}^{\alpha^{\left(j\right)}}\right\},$$
  and
  $$r^{\left(j\right)}\left(t\right)=\frac{\lambda^{\left(j\right)}\alpha^{\left(j\right)}{\left(\frac{t}{\beta^{\left(j\right)}}\right)}^{\alpha^{\left(j\right)}-1}\exp\left\{{\left(\frac{t}{\beta^{\left(j\right)}}\right)}^{\alpha^{\left(j\right)}}+\lambda^{\left(j\right)}\beta^{\left(j\right)}\left(1-\exp\left\{{\left(\frac{t}{\beta^{\left(j\right)}}\right)}^{\alpha^{\left(j\right)}}\right\}\right)\right\}}{1-\exp\left\{\lambda^{\left(j\right)}\beta^{\left(j\right)}\left(1-\exp\left\{{\left(\frac{t}{\beta^{\left(j\right)}}\right)}^{\alpha^{\left(j\right)}}\right\}\right)\right\}}.$$
6. Set j=j+1.
7. Repeat steps 2−5 *N* times.

Obtain the Bayes estimates of $\psi_{j}$ where $\psi_{1}=\alpha$, $\psi_{2}=\beta$, $\psi_{3}=\lambda$, $\psi_{4}=S\left(t\right)$, $\psi_{5}=h\left(t\right)$ and $\psi_{6}=r\left(t\right)$ for j=1,2,3,4,5 and 6 with respect to the SELF as

$$E(\psi_{j}|data)=\frac{1}{N-M}\sum_{i=M+1}^{N}\psi_{j}^{i}\text{ ,}\;$$

and under GELF as

$$E(\psi_{j}|data)={\left[\frac{1}{N-M}\sum_{i=M+1}^{N}{\left(\psi_{j}^{i}\right)}^{-\epsilon }\;\right]}^{\frac{-1}{\epsilon }}\;,\;\varepsilon \neq 0\;,$$

where *M* is the burn-in period. To establish the CRIs of $\psi_{j}$ order $\psi_{j}^{\left(M+1\right)}$, $\psi_{j}^{\left(M+2\right)},...,\psi_{j}^{\left(N\right)}$ and as $\psi_{j\left(1\right)}<$ $\psi_{j\left(2\right)}<...<\psi_{j\left(N-M\right)}$. Hence. The 100(1−2γ)% CRIs of $\psi_{j}$ can be constructed as

$$(\psi_{j\left(\gamma \left(N-M\right)\right)},\psi_{j\left(\left(1-\gamma \right)\left(N-M\right)\right)}).$$

## 4 Simulation study

Simulation studies play a pivotal role in the realm of statistical research, particularly in evaluating and comparing estimation methods. By simulating data under known conditions, researchers can systematically assess the performance of various statistical techniques across different scenarios. These studies provide a controlled environment where the true values are known, allowing for a rigorous comparison of estimation accuracy, precision, and robustness. Moreover, simulations facilitate the exploration of methodological assumptions and their implications in practical applications. They help identify strengths and weaknesses, guiding the selection of appropriate methods based on the specific characteristics of the data and research objectives. In essence, simulation studies serve as a cornerstone for advancing statistical methodologies, ensuring that researchers can confidently apply the most effective techniques to real-world data analysis challenges. Considering the suggested algorithm that Balakrishnan and Sandhu [31] with the CDF $1-{\left(1-F\left(x\right)\right)}^{k}$, 1000 PFFC samples were generated from MWD with the parameters (α,β,λ)=(1,0.1,2), k=2 and different (n,m). The performance of the derived estimates of *α*, *β* and *λ* from the proposed methods (MLE, Lindley approximation, MCMC technique) is compared in terms of point and interval estimates. To this end, the mean squared error, $MSE=\frac{1}{N}\sum_{i=1}^{N}{\left({\overset{ˆ}{\xi }}_{u}-\xi_{u}\right)}^{2}$ is considered for point estimates while the average confidence interval lengths (ACL)/credible interval lengths (CRIs) and coverage probability (CP) “the number of times the point estimate for the parameter falls within the estimated confidence interval” are considered for interval estimates. The results are shown in Table 1, Table 2, Table 3, Table 4, Table 5, Table 6, Table 7, Table 8. Bayes estimates and the CRIs are computed based on 12000 MCMC samples and discard the first values 2000 as “burn-in”, when the hyper-parameters are $a_{i}=1$ and $b_{i}=2,i=1,2$ and 3. In our study, various three censoring schemes are considered: Censoring scheme (SC) 1: $R_{1}=n-m$, $R_{i}=0$ for i≠1, SC 2: $R_{\frac{m}{2}}=R_{\frac{m}{2}+1}=\frac{n-m}{2}$, $R_{i}=0$ for $i\neq \frac{m}{2}$ and $i\neq \frac{m}{2}+1$ if *m* even; $R_{\frac{m+1}{2}}=n-m$, $R_{i}=0$ for $i\neq \frac{m+1}{2}$ if *m* odd. Finally, SC 3: $R_{m}=n-m$, for i≠m. From the results, several observations have emerged:

1. It is clear that from all Tables, as (n,m) increase, the MSEs decrease and the Bayes estimates under GELF with ϵ=1 have the smallest MSEs.
2. Scheme 1 performs better than other schemes in the sense of having smaller MSEs.
3. The MCMC CRIs give more accurate results than the ACIs because the lengths of the MCMC CRIs are smaller than the lengths of ACIs for different *n* and *m*.
4. Generally speaking, the Bayes estimates for the parameters using MCMC method are better than their MLEs and Bayes estimates using Lindley approximation, based on MSEs.
5. From Table 1, Table 2, Table 3, Table 4, Table 5, Table 6, Table 7, Table 8, the estimated values for all parameters using Lindley approximation under GELF at ϵ=−1 are exactly equal to the estimated values of all parameters using Lindley approximation under SELF.
6. From Table 1, Table 2, Table 3, Table 4, Table 5, Table 6, Table 7, Table 8, the estimated values for all parameters using MCMC under GELF at ϵ=−1 are exactly equal to the estimated values of all parameters using MCMC under SELF.
7. The estimates for the ML and Bayesian approaches are extremely similar, and their ACIs have high CPs.

**Table 1:** The MSE of the parameter *α*.

(*n*,*m*)	CS	MLE	Lindley			MCMC		
			SE	GE		SE	GE	
				*ϵ* = −1	*ϵ* = 1		*ϵ* = −1	*ϵ* = 1
(30,15)	1	0.34342	0.16173	0.16171	0.16023	0.07471	0.07473	0.07073
	2	0.34903	0.16784	0.16782	0.16472	0.07592	0.07596	0.07132
	3	0.34974	0.16815	0.16815	0.16482	0.07863	0.07864	0.07305
(30,20)	1	0.33782	0.14592	0.14594	0.15414	0.07206	0.07204	0.06564
	2	0.34181	0.15321	0.15323	0.15645	0.07424	0.07424	0.06695
	3	0.34273	0.15734	0.15736	0.15731	0.07464	0.07464	0.07042
(50,25)	1	0.30985	0.12583	0.12584	0.14023	0.06824	0.06822	0.05792
	2	0.32701	0.13266	0.13267	0.14786	0.06885	0.06885	0.05921
	3	0.33302	0.14214	0.14214	0.15072	0.07104	0.07105	0.06454
(50,40)	1	0.27831	0.12217	0.12217	0.12994	0.06394	0.06393	0.05296
	2	0.29546	0.12427	0.12427	0.13375	0.06625	0.06624	0.05479
	3	0.30344	0.12546	0.12548	0.13387	0.06812	0.06815	0.05547
(70,50)	1	0.25174	0.11735	0.11733	0.11013	0.05751	0.05759	0.05109
	2	0.26165	0.11625	0.11625	0.11392	0.05823	0.05824	0.05236
	3	0.26945	0.11524	0.11521	0.12044	0.0609	0.06094	0.05275
(90,75)	1	0.24305	0.10751	0.10751	0.10627	0.05454	0.05451	0.04302
	2	0.25025	0.10732	0.10732	0.10677	0.05476	0.05472	0.04553
	3	0.25153	0.11003	0.11002	0.10918	0.05678	0.05673	0.04831
(100,85)	1	0.22544	0.08569	0.08664	0.07569	0.03795	0.03886	0.03614
	2	0.22981	0.08897	0.08917	0.07725	0.03886	0.03947	0.03757
	3	0.23722	0.09543	0.09757	0.08946	0.04127	0.04195	0.03876

**Table 2:** The MSE of the parameter *β*.

(*n*,*m*)	CS	MLE	Lindley			MCMC		
			SE	GE		SE	GE	
				*ϵ* = −1	*ϵ* = 1		*ϵ* = −1	*ϵ* = 1
(30,15)	1	0.39661	0.17671	0.17674	0.17357	0.07471	0.07472	0.07792
	2	0.40472	0.18364	0.18367	0.17976	0.07675	0.07674	0.07973
	3	0.40772	0.18532	0.18534	0.18272	0.07952	0.07952	0.08272
(30,20)	1	0.37663	0.17193	0.17192	0.16884	0.05914	0.05913	0.07665
	2	0.39095	0.17336	0.17335	0.17224	0.05993	0.05995	0.07745
	3	0.39568	0.17427	0.17423	0.17304	0.06795	0.06797	0.07764
(50,25)	1	0.36797	0.13487	0.13483	0.14815	0.04832	0.04837	0.07211
	2	0.36943	0.15284	0.15287	0.14866	0.05361	0.05364	0.07265
	3	0.37216	0.16955	0.16954	0.16432	0.05764	0.05764	0.07596
(50,40)	1	0.33336	0.12576	0.12573	0.12574	0.04715	0.04711	0.05779
	2	0.36434	0.13003	0.13002	0.14075	0.04725	0.04723	0.06138
	3	0.36617	0.13087	0.13081	0.14756	0.04816	0.04815	0.06497
(70,50)	1	0.31215	0.11932	0.11934	0.12037	0.04209	0.04206	0.04634
	2	0.31396	0.12172	0.12175	0.12073	0.04318	0.04317	0.05165
	3	0.31764	0.12304	0.12304	0.12284	0.04324	0.04328	0.05186
(90,75)	1	0.26835	0.10723	0.10725	0.10676	0.03477	0.03478	0.03913
	2	0.28136	0.10862	0.10866	0.10747	0.03497	0.03495	0.03992
	3	0.28213	0.11884	0.11887	0.11892	0.03807	0.03804	0.04411
(100,85)	1	0.22843	0.09125	0.09328	0.09025	0.02998	0.03043	0.02871
	2	0.23541	0.10556	0.10673	0.10317	0.03253	0.03452	0.03115
	3	0.23895	0.10867	0.11028	0.10774	0.03342	0.03491	0.03213

**Table 3:** The MSE of the parameter *λ*.

(*n*,*m*)	CS	MLE	Lindley			MCMC		
			SE	GE		SE	GE	
				*ϵ* = −1	*ϵ* = 1		*ϵ* = −1	*ϵ* = 1
(30,15)	1	0.29942	0.17748	0.17742	0.16003	0.08182	0.08184	0.07378
	2	0.30485	0.18297	0.18292	0.16195	0.08204	0.08205	0.07495
	3	0.30543	0.18334	0.18334	0.17754	0.08217	0.08217	0.07894
(30,20)	1	0.28556	0.15957	0.15957	0.15204	0.07308	0.07305	0.06406
	2	0.28641	0.16003	0.16001	0.15623	0.07319	0.07312	0.06834
	3	0.29654	0.17622	0.17624	0.15675	0.07964	0.07961	0.07015
(50,25)	1	0.26787	0.14691	0.14697	0.14264	0.06525	0.06523	0.05316
	2	0.26794	0.15534	0.15535	0.14523	0.07096	0.07095	0.05467
	3	0.27335	0.15554	0.15556	0.14756	0.07301	0.07304	0.06217
(50,40)	1	0.25704	0.13846	0.13845	0.11997	0.05522	0.05524	0.04297
	2	0.26047	0.13955	0.13954	0.12157	0.05543	0.05547	0.04418
	3	0.26676	0.14157	0.14156	0.13774	0.06001	0.06004	0.05006
(70,50)	1	0.23782	0.11937	0.11937	0.11032	0.04502	0.04509	0.03504
	2	0.24457	0.13099	0.13097	0.11193	0.04813	0.04815	0.03802
	3	0.24644	0.13307	0.13304	0.11502	0.04894	0.04894	0.04211
(90,75)	1	0.23085	0.11268	0.11265	0.10562	0.04204	0.04206	0.03254
	2	0.23225	0.11328	0.11325	0.10724	0.04234	0.04235	0.03295
	3	0.23396	0.11397	0.11396	0.10805	0.04287	0.04284	0.03396
(100,85)	1	0.19459	0.09994	0.10437	0.09936	0.03858	0.03893	0.03757
	2	0.21115	0.10585	0.11109	0.10447	0.03999	0.04022	0.03955
	3	0.22015	0.11032	0.11237	0.10897	0.04119	0.04151	0.04074

**Table 4:** The ALs and CPs of 95% ACIs and CRIs for *α*, *β* and *λ*.

(*n*,*m*)	CS	MLE			MCMC		
		*α*	*β*	*λ*	*α*	*β*	*λ*
(30, 15)	1	5.54642	0.00981	9.55352	1.85912	0.000852	2.11682
		(0.9726)	(0.9318)	(0.9653)	(0.9268)	(0.9312)	(0.9408)
	2	5.71732	0.01002	9.71198	2.00981	0.000872	2.23992
		(0.9765)	(0.9375)	(0.9659)	(0.9272)	(0.9464)	(0.9534)
	3	5.90271	0.0103	10.68124	2.13674	0.000892	2.27943
		(0.9772)	(0.9385)	(0.9660)	(0.9278)	(0.9566)	(0.9549)
(30, 20)	1	5.31264	0.00724	9.01312	1.74293	0.00062	1.51881
		(0.9781)	(0.9387)	(0.9683)	(0.9305)	(0.9607)	(0.9581)
	2	5.32027	0.00744	9.37714	1.84405	0.000785	1.63272
		(0.9783)	(0.9381)	(0.9690)	(0.9313)	(0.9488)	(0.9591)
	3	5.51744	0.00901	9.39428	1.85008	0.000815	2.08042
		(0.9784)	(0.9403)	(0.9716)	(0.9314)	(0.9721)	(0.9723)
(50, 25)	1	5.17505	0.00586	8.65857	1.35944	0.000482	1.23638
		(0.9791)	(0.9416)	(0.9728)	(0.9359)	(0.9444)	(0.9634)
	2	5.20358	0.00606	8.74509	1.42318	0.000508	1.33817
		(0.9809)	(0.9424)	(0.9768)	(0.9365)	(0.9563)	(0.9669)
	3	5.26416	0.00713	8.88615	1.42782	0.000523	1.38501
		(0.9812)	(0.9435)	(0.9773)	(0.9366)	(0.9677)	(0.9699)
(50, 40)	1	4.36807	0.00506	6.63972	1.09021	0.000388	1.10087
		(0.9834)	(0.9437)	(0.9783)	(0.9377)	(0.9785)	(0.9719)
	2	4.44654	0.00555	7.18665	1.10485	0.000450	1.12642
		(0.9893)	(0.9474)	(0.9793)	(0.9389)	(0.9616)	(0.9726)
	3	4.94763	0.00565	7.96522	1.23286	0.000425	1.15444
		(0.9901)	(0.9490)	(0.9810)	(0.9429)	(0.9520)	(0.9750)
(70, 50)	1	4.10574	0.00276	5.72482	0.67922	0.000271	0.88804
		(0.9904)	(0.9513)	(0.9817)	(0.9460)	(0.9432)	(0.9789)
	2	4.18126	0.00375	6.32506	0.88905	0.000294	0.92334
		(0.9913)	(0.9514)	(0.9819)	(0.9461)	(0.9847)	(0.9737)
	3	4.33314	0.00391	6.36592	0.98852	0.000312	1.09232
		(0.9949)	(0.9524)	(0.9826)	(0.9467)	(0.9762)	(0.9839)
(90, 75)	1	3.23027	0.00181	4.89372	0.47425	0.000192	0.30392
		(0.9959)	(0.9538)	(0.9849)	(0.9504)	(0.9669)	(0.9879)
	2	3.24295	0.00242	5.30782	0.47542	0.000262	0.33475
		(0.9971)	(0.9582)	(0.9885)	(0.9507)	(0.9585)	(0.9910)
	3	3.67192	0.00261	5.38234	0.64976	0.00028	0.70745
		(0.9979)	(0.9606)	(0.9886)	(0.9509)	(0.9504)	(0.9970)

**Table 5:** The MSE of S(t) at *t* = 0.1.

(*n*,*m*)	CS	MLE	Lindley			MCMC		
			SE	GE		SE	GE	
				*ϵ* = −1	*ϵ* = 1		*ϵ* = −1	*ϵ* = 1
(30,15)	1	0.16802	0.18027	0.18027	0.17115	0.16977	0.16972	0.16562
	2	0.20435	0.19324	0.19324	0.18612	0.17861	0.17862	0.16893
	3	0.20973	0.20851	0.20854	0.20474	0.19511	0.19512	0.18443
(30,20)	1	0.15762	0.15154	0.15151	0.14320	0.12724	0.12725	0.10546
	2	0.16881	0.15904	0.15902	0.14807	0.14746	0.14745	0.13484
	3	0.17484	0.17266	0.17261	0.16344	0.15719	0.15717	0.15047
(50,25)	1	0.14357	0.10773	0.10774	0.10285	0.09376	0.09378	0.05897
	2	0.14607	0.12692	0.12695	0.11456	0.11343	0.11341	0.09382
	3	0.14871	0.13622	0.13622	0.11891	0.11694	0.11691	0.09822
(50,40)	1	0.10692	0.09981	0.09982	0.06731	0.06691	0.06692	0.04195
	2	0.11894	0.10417	0.10414	0.07721	0.06794	0.06793	0.04575
	3	0.12177	0.10719	0.10716	0.09531	0.07655	0.07654	0.04994
(70,50)	1	0.09148	0.08289	0.08287	0.04182	0.03585	0.03587	0.03242
	2	0.09868	0.08356	0.08357	0.05167	0.04886	0.04888	0.03555
	3	0.10191	0.09457	0.09453	0.06088	0.05171	0.05178	0.03861
(90,75)	1	0.04594	0.03824	0.03826	0.03818	0.01423	0.01426	0.01214
	2	0.06037	0.05265	0.05267	0.03047	0.03992	0.03995	0.01993
	3	0.07088	0.06274	0.06278	0.05067	0.03264	0.03264	0.02944
(100,85)	1	0.03445	0.02911	0.02999	0.02877	0.01165	0.01195	0.01025
	2	0.04255	0.03742	0.03854	0.03624	0.02567	0.02634	0.02416
	3	0.05864	0.05151	0.05267	0.04871	0.02896	0.02971	0.02757

**Table 6:** The MSE of h(t) at *t* = 0.1.

(*n*,*m*)	CS	MLE	Lindley			MCMC		
			SE	GE		SE	GE	
				*ϵ* = −1	*ϵ* = 1		*ϵ* = −1	*ϵ* = 1
(30,15)	1	0.42081	0.41794	0.41792	0.46783	0.35512	0.35512	0.34951
	2	0.42403	0.42101	0.42102	0.38574	0.36846	0.36843	0.35602
	3	0.43112	0.43025	0.43025	0.41476	0.39687	0.39684	0.36435
(30,20)	1	0.38074	0.35814	0.35815	0.32495	0.32324	0.32325	0.31762
	2	0.39685	0.37085	0.37087	0.33907	0.33695	0.33698	0.32869
	3	0.41504	0.40064	0.40062	0.36293	0.34653	0.34654	0.34345
(50,25)	1	0.31027	0.31045	0.31042	0.29313	0.27064	0.27065	0.20731
	2	0.36157	0.32434	0.32435	0.29516	0.27973	0.27971	0.22292
	3	0.37467	0.32632	0.32632	0.31743	0.31654	0.31659	0.24301
(50,40)	1	0.28972	0.24445	0.24443	0.20382	0.19861	0.19868	0.18672
	2	0.29433	0.25512	0.25515	0.22575	0.22234	0.22237	0.18832
	3	0.29874	0.29603	0.29606	0.24834	0.23324	0.23327	0.19314
(70,50)	1	0.25474	0.22025	0.22025	0.16532	0.14706	0.14702	0.13075
	2	0.28295	0.23533	0.23538	0.18027	0.16885	0.16885	0.15566
	3	0.28633	0.23793	0.23793	0.19432	0.17278	0.17271	0.16603
(90,75)	1	0.14216	0.12986	0.12985	0.12055	0.11841	0.11842	0.11356
	2	0.18302	0.13705	0.13705	0.13204	0.12312	0.12313	0.11805
	3	0.22401	0.20982	0.20985	0.16075	0.14293	0.14294	0.12484
(100,85)	1	0.12554	0.11624	0.11994	0.11233	0.09984	0.10245	0.99136
	2	0.14214	0.13522	0.12954	0.12104	0.11005	0.11546	0.10852
	3	0.17123	0.15117	0.15676	0.15015	0.13656	0.13977	0.12031

**Table 7:** The MSE of r(t) at *t* = 0.1.

(*n*,*m*)	CS	MLE	Lindley			MCMC		
			SE	GE		SE	GE	
				*ϵ* = −1	*ϵ* = 1		*ϵ* = −1	*ϵ* = 1
(30,15)	1	0.43803	0.43332	0.43335	0.41061	0.40361	0.40367	0.38978
	2	0.45856	0.45131	0.45137	0.43372	0.43072	0.43075	0.41256
	3	0.46322	0.45845	0.45848	0.45583	0.45503	0.45504	0.44525
(30,20)	1	0.40911	0.40557	0.40554	0.36867	0.34746	0.34744	0.32962
	2	0.42964	0.42637	0.42635	0.37654	0.36534	0.36535	0.33885
	3	0.43707	0.42767	0.42762	0.41022	0.38557	0.38554	0.36527
(50,25)	1	0.37288	0.34943	0.34941	0.31943	0.30085	0.30087	0.24177
	2	0.37633	0.36916	0.36912	0.32892	0.32508	0.32503	0.27617
	3	0.39134	0.37855	0.37855	0.33365	0.34441	0.34442	0.28483
(50,40)	1	0.27705	0.27145	0.27147	0.26201	0.21332	0.21334	0.19756
	2	0.31541	0.28467	0.28466	0.26862	0.22763	0.22761	0.21985
	3	0.32104	0.32055	0.32055	0.28133	0.28016	0.28014	0.23168
(70,50)	1	0.24342	0.22584	0.22582	0.19826	0.16744	0.16747	0.14552
	2	0.26005	0.25664	0.25667	0.23035	0.18337	0.18337	0.14595
	3	0.26671	0.26503	0.26504	0.23705	0.19454	0.19456	0.16983
(90,75)	1	0.19424	0.15241	0.15241	0.12664	0.11782	0.11787	0.11159
	2	0.19502	0.16214	0.16214	0.14665	0.13922	0.13928	0.12129
	3	0.23415	0.20266	0.20261	0.15546	0.15513	0.15514	0.12874
(100,85)	1	0.16237	0.13455	0.13661	0.11473	0.09875	0.10239	0.09744
	2	0.18567	0.14445	0.15013	0.14232	0.11824	0.11994	0.11024
	3	0.19997	0.17593	0.17692	0.16874	0.13227	0.13943	0.11875

**Table 8:** The ALs and CPs of 95% ACIs and CRIs for S(t), h(t) and r(t) at *t* = 0.1.

(*n*,*m*)	CS	MLE			MCMC		
		S(t)	h(t)	r(t)	S(t)	h(t)	r(t)
(30, 15)	1	10.10082	4.32232	2.93552	8.03972	2.70723	2.25274
		(0.9523)	(0.9452)	(0.9634)	(0.9554)	(0.9757)	(0.9621)
	2	5.79164	3.24542	2.68913	6.69564	3.18315	2.82436
		(0.9529)	(0.9649)	(0.9728)	(0.9547)	(0.9649)	(0.9669)
	3	5.05756	4.13545	2.71344	8.71494	6.87245	1.70287
		(0.9649)	(0.9448)	(0.9398)	(0.9559)	(0.9475)	(0.9499)
(30, 20)	1	8.94574	2.34414	2.64623	6.18393	1.55515	1.45165
		(0.9492)	(0.9594)	(0.9495)	(0.9591)	(0.9697)	(0.9594)
	2	6.55635	2.60644	2.16595	5.94386	1.95433	1.29714
		(0.9397)	(0.9459)	(0.9396)	(0.9479)	(0.9519)	(0.9598)
	3	5.94754	2.53435	2.16995	6.97781	2.77412	1.25672
		(0.9419)	(0.9456)	(0.9505)	(0.9529)	(0.9618)	(0.9639)
(50, 25)	1	5.83944	1.55943	1.73295	4.78995	1.20574	1.06946
		(0.9459)	(0.9528)	(0.9619)	(0.9558)	(0.9609)	(0.9547)
	2	3.99915	1.96318	1.95359	4.45992	1.77552	1.87563
		(0.9518)	(0.9549)	(0.9496)	(0.9478)	(0.9579)	(0.9489)
	3	3.92234	2.43615	2.05875	5.86443	3.43262	1.75212
		(0.9505)	(0.9398)	(0.9486)	(0.9419)	(0.9399)	(0.9456)
(50, 40)	1	5.82644	1.28834	1.49794	3.98916	1.72948	1.62027
		(0.9619)	(0.9658)	(0.9714)	(0.9669)	(0.9599)	(0.9498)
	2	4.17687	1.34514	1.25334	3.83223	2.50642	1.52985
		(0.9541)	(0.9524)	(0.9611)	(0.9574)	(0.9499)	(0.9510)
	3	6.67064	2.69944	2.48853	5.35245	2.10485	1.59182
		(0.9399)	(0.9478)	(0.9426)	(0.9547)	(0.9468)	(0.9481)
(70, 50)	1	5.57781	2.44396	2.50695	4.78375	1.78584	1.62094
		(0.9421)	(0.9399)	(0.9432)	(0.9345)	(0.9454)	(0.9373)
	2	3.83227	2.21991	2.25311	4.31552	2.19053	1.45785
		(0.9565)	(0.9459)	(0.9456)	(0.9532)	(0.9479)	(0.9533)
	3	5.47212	2.42082	2.82712	5.03792	2.52617	1.55768
		(0.9499)	(0.9577)	(0.9721)	(0.9557)	(0.9469)	(0.9476)
(90, 75)	1	4.75732	1.60632	2.36044	3.95474	1.34514	1.28465
		(0.9329)	(0.9634)	(0.9457)	(0.9810)	(0.9753)	(0.9647)
	2	4.60335	1.59657	2.25646	3.79954	1.40746	1.22816
		(0.9559)	(0.9547)	(0.9608)	(0.9619)	(0.9599)	(0.9548)
	3	5.64526	1.64415	2.08844	4.27816	1.55035	1.24094
		(0.9619)	(0.9598)	(0.9589)	(0.9601)	(0.9557)	(0.9643)

## 5 Application on renal transplant survival times

To elucidate the estimating methodologies covered in the preceding sections We offer an application of real-world data for renal transplant survival times. An actual data set was first reported by Hand et al. [32]. The information shows the graft survival times (in years) for one hundred kidney transplant recipients. The data is listed as follows: 0.0035, 0.0068, 0.0101, 0.0167, 0.0168, 0.0197, 0.0213, 0.0233, 0.0234, 0.0508, 0.0508, 0.0533, 0.0633, 0.0767, 0.0768, 0.0770, 0.1066, 0.1267, 0.1300, 0.1639, 0.1803, 0.1867, 0.2180, 0.2967, 0.3328, 0.3700, 0.3803, 0.4867, 0.6233, 0.6367, 0.6600, 0.7180, 0.7800, 0.7967, 0.8016, 0.8300, 0.8410, 0.9100, 0.9233, 1.0541, 1.0607, 1.0633, 1.1067, 1.2213, 1.2508, 1.2533, 1.3800, 1.4267, 1.4475, 1.4500, 1.5213, 1.5333, 1.5525, 1.5533, 1.5541, 1.5934, 1.6200, 1.6300, 1.6344, 1.6600, 1.7033, 1.7067, 1.7475, 1.7667, 1.7700, 1.7967, 1.8115, 1.8933, 1.8934, 1.9508, 1.9733, 2.0180, 2.0900, 2.1167, 2.1233, 2.2100, 2.2148, 2.2267, 2.2500, 2.2533, 2.3738, 2.4082, 2.418, 2.4705, 2.5213, 2.5705, 3.1934, 3.2180, 3.2367, 3.2705, 3.3148, 3.3567, 3.4836, 3.4869, 3.6213, 3.9410, 3.9433, 4.0001, 4.1733, 4.1734.

**Figure 3:**
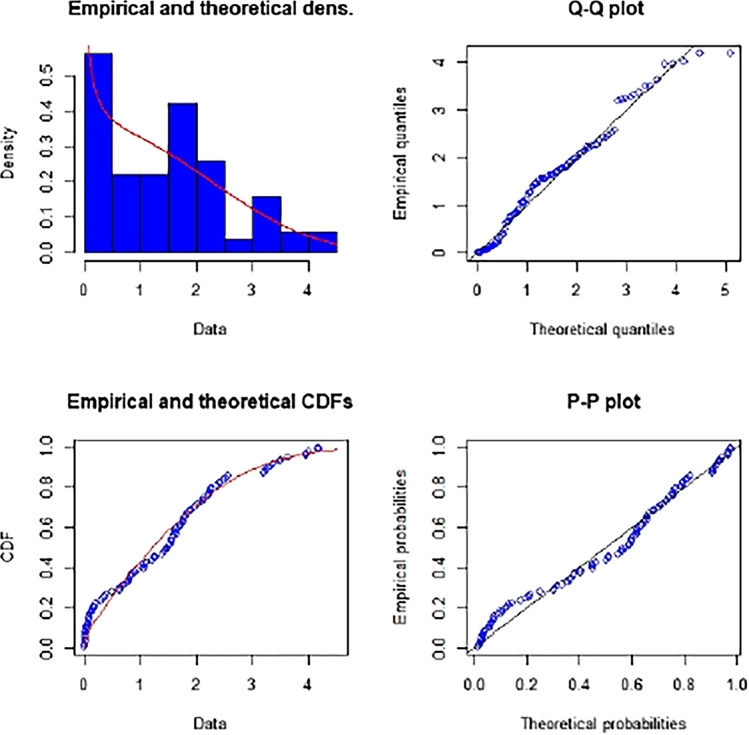
The fitting plots for the MWD.

The Kolmogorov-Smirnov (K-S) test statistic is used to assess the degree of fit between the MWD and the actual data. The K-S distances and associated p-value are calculated and come out to be 0.3571 and 0.092661, respectively. Based on the p-value, we can conclude that the MWD fits the data exactly. Fig. 3 provides additional illustrations in the form of empirical, Q-Q, and P-P charts.

According to the above data set, a PFFC sample of size m=10 is generated. The data is randomly divided into n=20 groups with k=5 units in each group. Suppose that the pre-defined scheme is R={2,1,1,2,1,1,1,1,0,0}. Then, the PFFC sample generated from the above data is 0.0035, 0.0068, 0.0101, 0.0168, 0.0213, 0.0234, 1.1067, 2.018, 2.2267, 3.1934.

**Figure 4:**
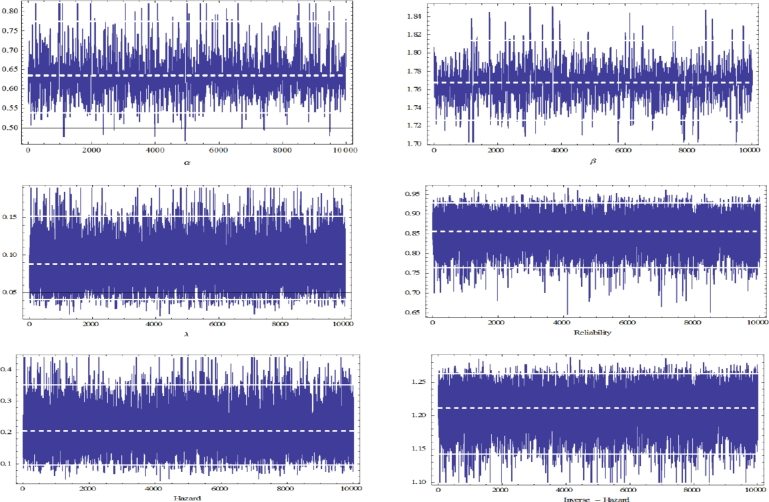
Trace plots of *α*, *β*, *λ*, reliability, hazard, and inverse hazard obtained from MCMC.

**Figure 5:**
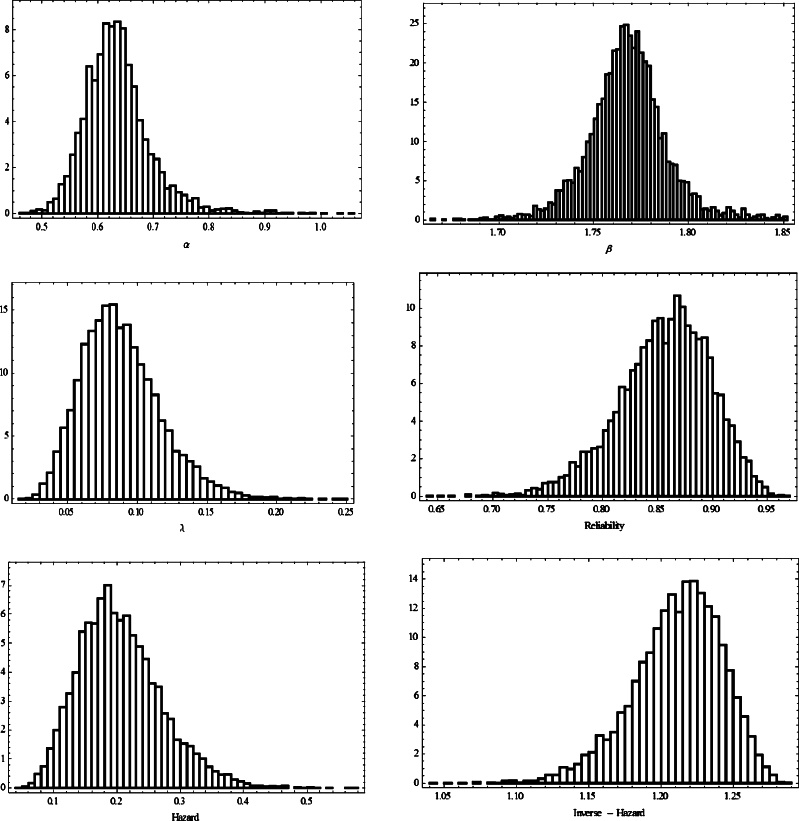
Histograms of *α*, *β*, *λ*, reliability, hazard, and inverse hazard obtained from MCMC.

Based on the previous sample of PFFC, the MLEs and ACIs for *α*, *β*, *λ*, S(t), h(t) and r(t) are determined to be as in Table 9, Table 10. Moreover, to compute the Bayesian estimates, the prior distributions of the parameters are needed to specify. Since we have no prior information, we assume that the non-informative gamma priors for *α*, *β* and *λ* that is, when the hyper-parameters are $a_{i}=0.0001$ and $b_{i}=0.0001$, i=1,2,3. In addition, 12000 MCMC samples were generated and the first 2000 samples expunged as ‘burn-in’. Figure 4, Figure 5 display the trace plots of the parameters generated by the MCMC approach and the associated histograms. The dashed line to verify the convergence of the MCMC method (around point estimation of the parameter). While the solid line determines the lower and upper bounds of the credible intervals. Table 9, Table 10 show the Bayesian estimates as well as 95% CRIs for *α*, *β*, *λ*, S(t), h(t) and r(t).

**Table 9:** Point estimates of *α*, *β*, *λ*, S(t), h(t) and r(t).

Parameter	MLE	Bayes					
		Lindley			MCMC		
		SE	GE		SE	GE	
			*ϵ* = −1	*ϵ* = 1		*ϵ* = −1	*ϵ* = 1
*α*	0.65122	0.64074	0.64675	0.63222	0.63525	0.63748	0.63393
*β*	1.77911	1.77215	1.78136	1.77023	1.76814	1.76745	1.76126
*λ*	0.09011	0.08876	0.08993	0.08684	0.08715	0.08812	0.08697
S(1)	0.87912	0.86075	0.86084	0.85944	0.85413	0.85421	0.85315
h(1)	0.22073	0.21653	0.21964	0.21253	0.21646	0.20644	0.20394
r(1)	1.21844	1.21334	1.21535	1.20115	1.21227	1.21557	1.21183

**Table 10:** 95% ACIs and CRIs of *α*, *β*, *λ*, S(t), h(t) and r(t).

Parameter	ACI		CRI	
	Interval	Length	Interval	Length
*α*	[0.4957,0.7105]	0.2148	[0.5412,0.7258]	0.1846
*β*	[1.6645,1.8749]	0.2104	[1.7221,1.8153]	0.0932
*λ*	[0.0447,0.1766]	0.1319	[0.0321,0.1524]	0.1203
S(1)	[0.6399,0.9189]	0.2790	[0.7653,0.9285]	0.1632
h(1)	[0.1002,0.4212]	0.3210	[0.0997,0.3554]	0.2557
r(1)	[1.0997,1.2701]	0.1704	[1.1436,1.2637]	0.1201

## 6 Conclusion

In this study, we have devised three different methods employing a PFFC scheme to estimate the unknown parameters of the MWD. Using the Fisher information matrix, we have created ACIs for *α*, *β* and *λ*. Furthermore, the ACIs for SF, HRF, and IHRF have been computed using the delta approach. It is clear that the posterior distribution equations for the unknown parameters are complex and difficult to reduce analytically into well-known forms, particularly when taking Bayesian estimates into account. We have used MCMC techniques and the Lindley approximation to compute the Bayesian estimators in order to overcome this difficulty. We have calculated these Bayes estimates for both SELF and GELF. In addition, the study began by evaluating various methodologies and directly comparing their performance in a simulated environment. Based on the results obtained, it was determined that the Bayes method is suitable for estimating and constructing approximate confidence intervals for unknown parameters when dealing with progressively first-failure censored data from the MWD. Furthermore, the MCMC algorithm demonstrated superior performance compared to Lindley's method. Subsequently, the MWD was applied to real-world medical data, revealing its capability to accurately model current data, thereby suggesting its potential for analyzing similar datasets in the medical field. Despite these findings, the study highlights several avenues for future research. Specifically, optimizing censoring schemes for enhanced effectiveness and extending statistical inference methods to accommodate accelerated life testing models with multiple failure factors remain important areas for future investigation. Also, our paper can have many impacts and benefits across different fields we list it as follows:

• Healthcare and Biomedical Research: In healthcare, understanding the distribution of extreme medical events, such as patient survival times or disease progression, is vital for treatment planning and resource allocation. By applying the modified Weibull distribution in a first-failure censored progressive approach, researchers can better model and analyze medical data, leading to advancements in treatment strategies and patient care.
• Engineering and Reliability Analysis: This research can significantly impact industries where reliability analysis is crucial, such as aerospace, automotive, and manufacturing. By accurately modeling extreme data using the modified Weibull distribution, engineers can better understand the failure mechanisms of components and systems. This understanding can lead to improved designs, maintenance strategies, and product reliability.
• Statistical Methodology: The development of new statistical methods for analyzing extreme data has broader implications for the field of statistics itself. Researchers and practitioners in statistics and data science can benefit from the theoretical framework and inference techniques proposed in this research, potentially leading to advancements in other areas of statistical modeling and inference.

## CRediT authorship contribution statement

**Mohamed S. Eliwa:** Writing – review & editing, Visualization, Formal analysis, Data curation, Conceptualization. **Laila A. Al-Essa:** Writing – review & editing, Resources, Methodology, Funding acquisition. **Amr M. Abou-Senna:** Writing – original draft, Validation, Software, Data curation. **Mahmoud El-Morshedy:** Validation, Software, Resources, Conceptualization. **Rashad M. EL-Sagheer:** Writing – original draft, Visualization, Methodology, Data curation, Conceptualization.

## Declaration of Competing Interest

The authors declare that they have no known competing financial interests or personal relationships that could have appeared to influence the work reported in this paper.

## Data Availability

The data that supports the findings of this study are available within the article.
